# Supplementary material for: Rapid Sample Screening Method for Authenticity Controlling of Vanilla Flavours Using Liquid Chromatography with Electrochemical Detection Using Aluminium-Doped Zirconia Nanoparticles-Modified Electrode
Source: Molecules. 2022 May 3;27(9):2915. doi: 10.3390/molecules27092915 (PMC9105493; doi:10.3390/molecules27092915)
Supplement: Supplementary file 1 [file molecules-27-02915-s001.zip › molecules-1701528-supplementary.pdf]

Electronic Supplementary Materials

Rapid Sample Screening Method for Authenticity Controlling of Vanilla Flavours Using Liquid Chromatography with Electrochemical Detection Using Aluminium-Doped Zirconia Nanoparticles-Modified Electrode

Yassine Benmassaoud [1,2,3], Khaled Murtada [1,2,†], Rachid Salghi [3], Mohammed Zougagh [1,2] and Ángel Ríos [1,2,*]

[1] Department of Analytical Chemistry and Food Technology, Faculty of Pharmacy, University of Castilla-La Mancha, 13071 Ciudad Real, Spain; yassinebenmassaoud@gmail.com (Y.B.); kmurtada@uwaterloo.ca (K.M.); mohammed.zougagh@uclm.es (M.Z.)

[2] Analytical-NANO-Group, Regional Institute for Applied Chemistry Research (IRICA),
13071 Ciudad Real, Spain

[3] Laboratory of Applied Chemistry and Environment, Ecole Nationale Des Sciences Appliquées (ENSA), Université Ibn Zohr, P.O. Box 1136, Agadir 80000, Morocco; r.salghi@uiz.ac.ma

* Correspondence: angel.rios@uclm.es

† Current Address: Department of Chemistry, University of Waterloo, 200 University Avenue West,
Waterloo, ON N2L 3G1, Canada.

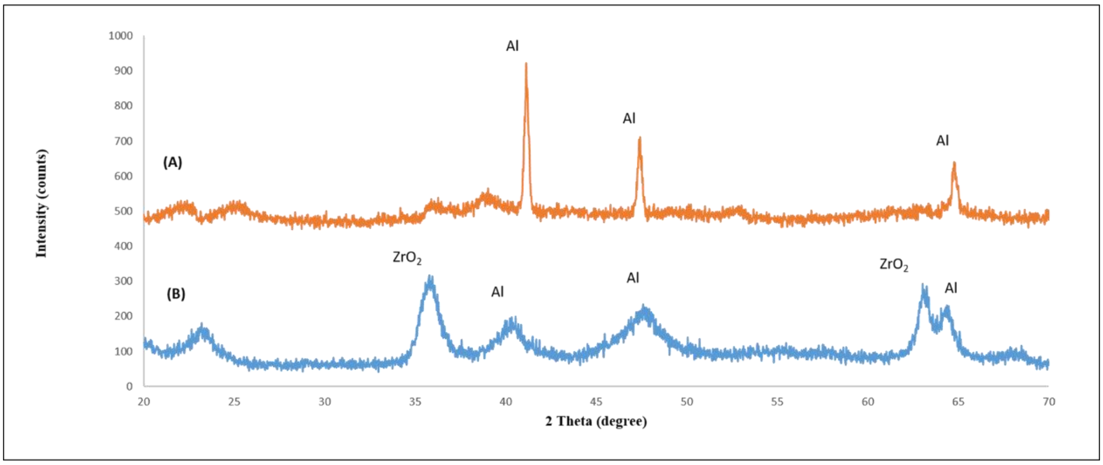

Figure S1. XRD patterns measured for (A) Al-NPs and (B) Al-ZrO_2-NPs.

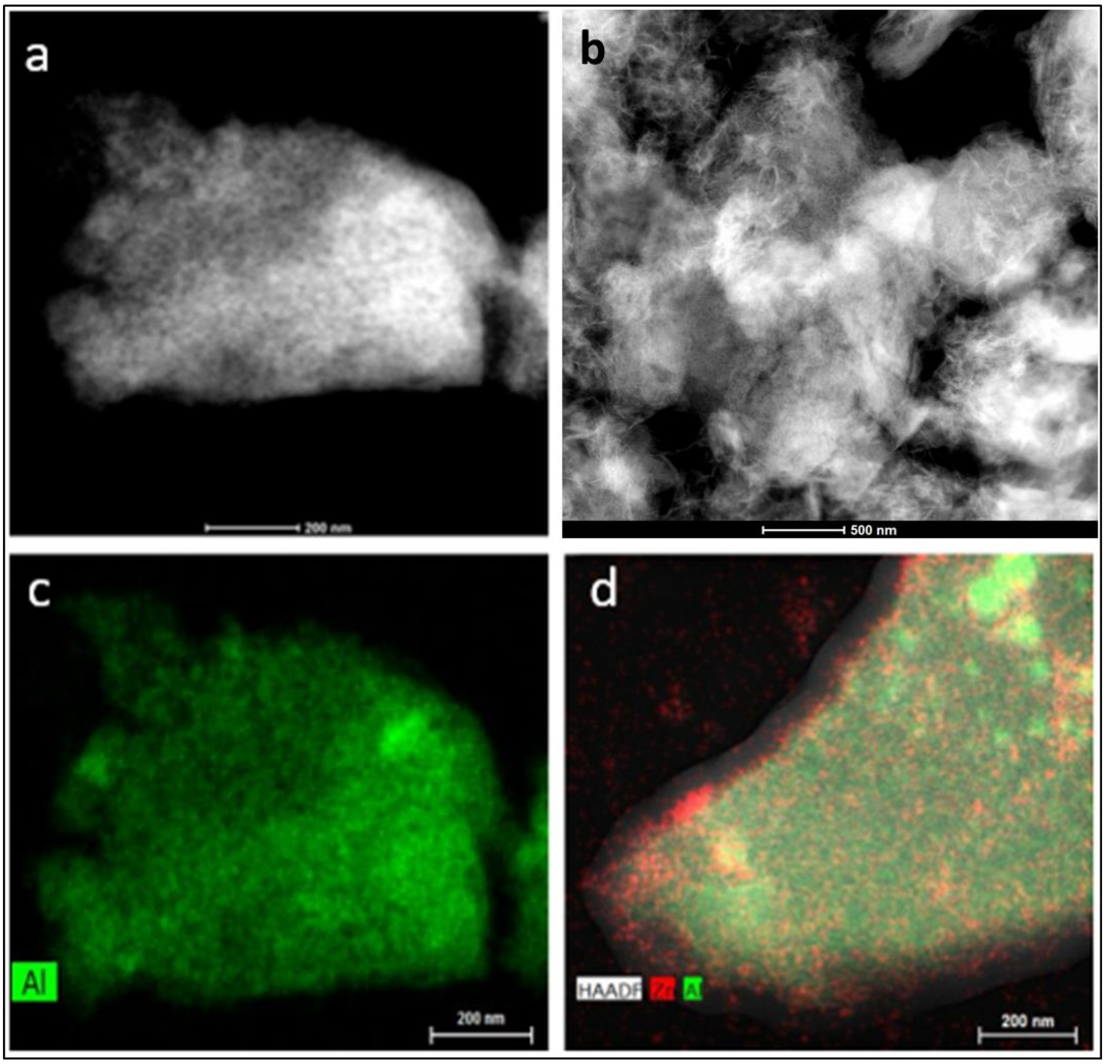

Figure S2. TEM and EDX micrographs of Al-NPs (a and c) and Al-ZrO_2-NPs (b and d).

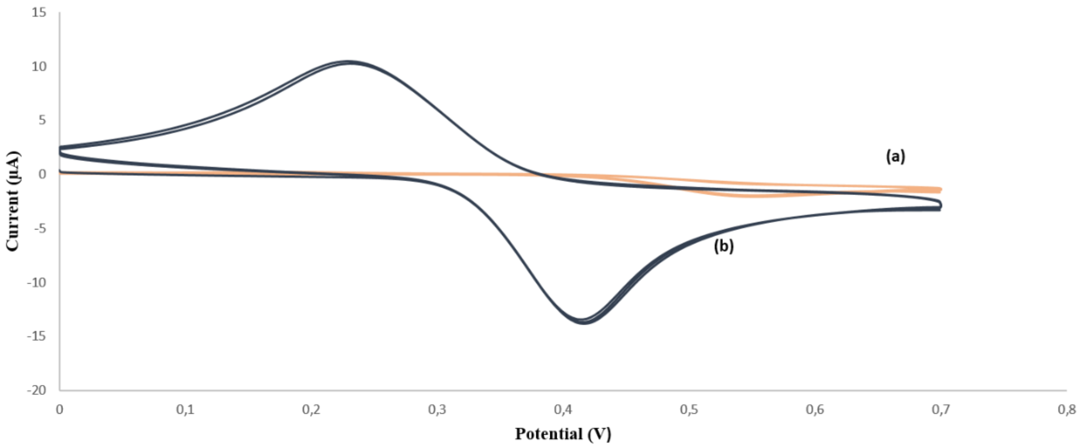

Figure S3. The cyclic voltammograms of 0.1 mM of dopamine in phosphoric acid solution (0.1 M) as electrolyte, 50 mV/s scan rate. (a) SPCE and (b) Al-ZrO_2-NPs/SPCE.
